# Antibiotic prescribing patterns and carriage of antibiotic-resistant *Escherichia coli* and *Enterococcus* species in healthy individuals from selected communities in Lusaka and Ndola districts, Zambia

**DOI:** 10.1093/jacamr/dlae027

**Published:** 2024-03-05

**Authors:** Kaunda Yamba, Steward Mudenda, Evans Mpabalwani, Geoffrey Mainda, Mercy Mukuma, Mulemba Tillika Samutela, Chileshe Lukwesa, Joseph Chizimu, Ciluvya Kavimba Kaluba, Matenge Mutalange, Roma Chilengi, John Bwalya Muma

**Affiliations:** Department of Pathology & Microbiology, University Teaching Hospitals, Lusaka, Zambia; Department of Disease Control University of Zambia, School of Veterinary Medicine, University of Zambia, Lusaka, Zambia; Antimicrobial Resistance Cluster, Zambia National Public Health Institute, Lusaka, Zambia; Department of Disease Control University of Zambia, School of Veterinary Medicine, University of Zambia, Lusaka, Zambia; Department of Pharmacy, School of Health Sciences, University of Zambia, Lusaka, Zambia; Department of Paediatrics & Child Health, School of Medicine, University of Zambia, Lusaka, Zambia; Food and Agriculture Organization (FAO) of the United Nations, House No. 5, Chaholi, Off Addis Ababa Drive, Lusaka, Zambia; Department of Veterinary Services Central Veterinary Research Institute (CVRI), Ministry of Fisheries and Livestock, Lusaka, Zambia; Department of Food Science, School of Agricultural Sciences and Nutrition, University of Zambia, Lusaka, Zambia; Department of Biomedical Sciences, School of Health Sciences, University of Zambia, Lusaka, Zambia; Department of Pathology & Microbiology, University Teaching Hospitals, Lusaka, Zambia; Antimicrobial Resistance Cluster, Zambia National Public Health Institute, Lusaka, Zambia; Department of Disease Control University of Zambia, School of Veterinary Medicine, University of Zambia, Lusaka, Zambia; Department of Disease Control University of Zambia, School of Veterinary Medicine, University of Zambia, Lusaka, Zambia; Department of Pathology and Microbiology, School of Medicine and Health Sciences, Mulungushi University, Livingstone, Zambia; Zambia National Public Health Institute, Ministry of Health, Lusaka, Zambia; Department of Disease Control University of Zambia, School of Veterinary Medicine, University of Zambia, Lusaka, Zambia

## Abstract

**Objectives:**

This study assessed antibiotic prescribing patterns in primary healthcare facilities and antimicrobial resistance (AMR) profiles of commensal *Escherichia coli* and enterococci isolated from pregnant women and children under 5 years of age.

**Materials and methods:**

This cross-sectional study was conducted in Lusaka and Ndola districts of Zambia. Prescription pattern data were obtained from hospital pharmacies. Identification and antimicrobial susceptibility profiles of *E. coli* and enterococci were determined by conventional methods, while confirmation of both pathogens and AMR genes were determined by PCR. Data were analysed using WHONET and SPSS version 25.0.

**Results:**

Most prescribed antibiotics at the primary healthcare facilities belonged to the Access group of the WHO Access, Watch and Reserve (AWaRe) classification. All the primary healthcare facilities adhered to the AWaRe framework of ≥60% prescribed antibiotics belonging to the Access group. However, resistance was highest in the Access group of antibiotics. *E. coli* resistance to ampicillin ranged from 71% to 77% and to co-trimoxazole from 74% to 80%, while enterococcal resistance to tetracycline was 59%–64%. MDR was highest in *E. coli* (75%) isolates, while XDR was highest in enterococcal isolates (97%). The identified AMR genes in *E. coli* included *bla*_CTX-M_, *sul*2 *and qnr*A, while those of enterococci included *erm*(B), *erm*(C) and *erm*(A).

**Conclusions:**

Resistance was highest in the prescribed WHO Access group of antibiotics. These findings highlight the need to use local susceptibility data to formulate country-specific treatment guidelines in line with WHO AWaRe classification and enforce regulations that prohibit easy access to antibiotics.

## Introduction

The human gut microbiota harbours both commensals and opportunistic pathogens, which can acquire antibiotic resistance through mutation and horizontal gene transfer.^1^ The term ‘human gut microbiota’ describes the microbial community colonizing the human intestinal tract, which has a symbiotic relationship with its host, facilitating a balanced, mutually beneficial state.^2^ The gut microbiota maintains human nutrition and health by supplying nutrients and providing protection from pathogenic organisms.^3^ When pathogenic bacteria invade the host, the intestine’s immune system can distinguish commensal bacteria from pathogenic ones and only attack those harmful to the host.^4^ Equally, opportunistic pathogens carried asymptomatically in healthy individuals can also be present in the gut microbiota and only cause infections in immunocompromised hosts.^1^

*Escherichia coli* and enterococci are the most prevalent commensal bacteria that colonize the gastrointestinal tract (GIT) of humans and animals and are also known to be opportunistic pathogens.^5^ Both *E. coli* and enterococci are causative agents of community-acquired and healthcare-associated infections, with *E. coli* being the leading cause of bloodstream infections (BSIs) and urinary tract infections (UTIs).^6,7^ The outcome of infections caused by these pathogens has become difficult to treat due to the emergence of antimicrobial resistance (AMR).^8^ The escalating predominance of MDR organisms (MDROs) in the community and the increasing incidence of community-associated AMR infections pose a significant threat to public health.^9,10^ AMR in commensal bacteria could contribute to an increase in AMR among pathogenic bacteria through the horizontal transfer of resistance genes.^11^ This cross-transmission of AMR genes from commensal to pathogenic bacteria and vice versa may lead to community- or hospital-acquired infections caused by resistant pathogens, should such bacteria acquire additional genetic material that enables them to become pathogenic.^12^

Most primary healthcare facilities in low- and middle-income countries (LMICs) lack microbiology diagnostic infrastructure and capacity; hence, treatment is often empirical without microbiology guidance.^13–15^ Treating infections without the guidance of antimicrobial susceptibility coupled with the purchase of antibiotics without clinical indication and prescriptions worsens the emergence and spread of AMR in the community and further limits treatment options for more invasive infections.^16,17^ The WHO has developed a framework for antimicrobial stewardship; this tool guides antimicrobial usage and limits the selection and spread of antibiotic resistance.^18^ This tool emphasizes that narrow-spectrum antibiotics included in the Access group should be preferred over broad-spectrum antibiotics from the Watch and Reserve groups and encourages a target of at least 60% of total antibiotic consumption to be from the Access group.^19,20^ However, there have been inconsistencies in adherence to this framework due to the lack of locally generated antimicrobial susceptibility data and sustained, reliable availability of antibiotics that supports the allocation of antibiotics in the Access, Watch and Reserve (AWaRe) classification.^21,22^

The human gut microbiota has been identified as a reservoir of AMR genes, referred to as the gut resistome, thus there is a need to study antibiotic resistance in the human gut microbiota to characterize the resistome’s ability to contribute to the emergence of MDR opportunistic pathogens.^1^ Knowledge of resistance patterns and the burden of MDR among commensal bacteria could help predict the resistance profile of a subsequent clinical infection.^12^ Resistance genes are prevalent in the faecal *E. coli* strains from healthy individuals; hence, AMR surveillance programmes have highlighted the importance of assessing resistance patterns in commensal intestinal bacteria to estimate AMR trends in the communities.^23,24^ It is in this regard that this study sought to assess the antibiotic prescribing patterns and AMR profiles of commensal *E. coli* and enterococci isolated from healthy pregnant women and children ≤5 years old who accessed antenatal and under-five services at the selected primary healthcare facilities in Lusaka and Ndola districts of Zambia.

## Materials and methods

### Study design and site

This cross-sectional study was conducted in the Lusaka and Ndola districts of Zambia between October 2020 and January 2021. Rectal swabs were collected from pregnant women and children ≤5 years old from different communities attending antenatal and under-five care, respectively, at primary healthcare facilities in Lusaka and Ndola. Inclusion criteria were all pregnant women and children ≤5 years old whose mothers gave consent and had no history of hospital admission and/or antibiotic use in the past 30 days.

#### Study area in Lusaka district

Three primary healthcare facilities targeted in the Lusaka district were Chilenje and Kanyama first-level hospitals and Kalingalinga health centre. Kanyama is densely populated with approximately 169 253 inhabitants, and a population density of 5636/km^2^.^25^ Kalingalinga and Chilenje are middle-density areas with approximately 39 139 and 52 220 inhabitants, respectively, and 3771 and 4769/km^2^ population density, respectively.^25^

#### Study area in Ndola district

Similarly, the three primary healthcare facilities targeted in the Ndola district were Lubuto, New Masala and Mapalo health centres. Lubuto has a population of 22 915 and 7695/km^2^ population density, New Masala has 9059 inhabitants and 10 391/km^2^ population density, and Mapalo urban health centres have 37 703 inhabitants and 7769/km^2^.^25^

All six healthcare facilities acted as the first contact point for patients, offering both inpatient and outpatient services to the approximated inhabitants of their catchment area. However, cases needing specialist care were transferred to tertiary hospitals. A tertiary hospital is a hospital that provides healthcare obtained from specialists in a large hospital after referral from the providers of primary care and secondary care.

### Data collection

#### Antibiotic prescribing patterns

Antibiotic prescribing patterns were collected from the six primary healthcare facilities using a questionnaire imbedded in the CSPro 7.6 data collection tool (https://cspro.software.informer.com/7.6/) and exported into Excel, where the antibiotics were categorized as ‘Access’, ‘Watch’ or ‘Reserve’ according to the WHO AWaRe classification of antibiotics.^26^

#### Specimen collection and processing

After obtaining consent and explaining the procedure to the participants, a sterile swab moistened in 0.85% sterile saline was used to collect a rectal swab. The swab was placed into Amie’s transport medium (Oxoid, Basingstoke, UK). Upon receipt at the Veterinary Public Health Laboratory at the University of Zambia, the swabs were immediately placed in alkaline peptone water (APW) and incubated aerobically at 35–37°C for 18–24 h before subculturing. A total of 290 rectal swabs were collected from pregnant women and children under 5 years, of which 168 were collected in Lusaka and 122 in Ndola.

#### Identification of E. coli

The samples were then cultured on MacConkey agar (Oxoid, Basingstoke, UK) and incubated aerobically at 35°C–37°C for 18–24 h. Bacterial growth on the plates was examined for colony morphology characteristic of *E. coli*. Gram stain was also performed to confirm Gram-negative morphology. Presumptive identification of *E. coli* was based on standard biochemical reactions while PCR was used to confirm the species identification of *E. coli*. This was achieved using the *uidA* gene, which encodes the β-glucuronidase enzyme.^27^ DNA was extracted using the NucliSENS Easy MAG machine (bioMérieux). PCR amplification was performed as described by Godambe *et al.*^27^ The Veriti 96 Well Thermal Cycler-Applied Biosystems (Pittsburgh, PA, USA) was used for PCR amplification. The PCR products (1/10 volume) were analysed by gel electrophoresis (Bio-Rad, Hercules, CA, USA) at 100 V for 30 min using 1.5% agarose gels (BD Difco) in 1× Tris-acetate EDTA (TAE) buffer. The gels were stained with ethidium bromide (Sigma, St. Louis, MO, USA), and the PCR products were visualized under UV light using a gel documentation machine.

### Identification of Enterococcus species

The samples were cultured on blood agar (Oxoid, Basingstoke, UK) and then incubated in a 5% CO_2_ incubator at 35°C–37°C for 18–24 h. Bacterial growth on the plates was examined for colony morphology characteristic of enterococci. Gram stain was performed, and the Gram-positive cocci and catalase-negative isolates were then plated on bile esculin azide (BEA) agar (Oxoid, Basingstoke, UK) to identify enterococci presumptively. PCR was conducted on single colonies that turned the media black (bile aesculin-positive colonies) for genus confirmation using the *tuf* gene, while the *sodA* gene was used to speciate *Enterococcus faecium* and *Enterococcus faecalis*, respectively, as was done by Li *et al.*^28^ and Pillay *et al.*^29^

### Antimicrobial susceptibility testing (AST) of E. coli and Enterococcus species

Conventional AST using the Kirby–Bauer disc diffusion method was used to determine antimicrobial susceptibility profiles of *E. coli* and enterococci. The following antibiotics were used. For *E. coli:* ampicillin 10 μg, cefoxitin 30 μg, cefotaxime 30 μg, ceftazidime 30 μg, cefepime 30 μg, imipenem 10 μg, meropenem 10 μg, co-trimoxazole 1.25 μg/23.75 μg, ciprofloxacin 5 μg, gentamicin 10 μg, amikacin 30 μg, tetracycline 30 μg, nitrofurantoin 300 μg and aztreonam 30 μg; for enterococci: high-level gentamicin 120 μg, chloramphenicol 30 μg, vancomycin 30 μg, erythromycin 15 μg, nitrofurantoin 300 μg, ampicillin 10 μg, penicillin 10 μg, ciprofloxacin 5 μg, quinupristin/dalfopristin 15 μg, tetracycline 30 μg, linezolid 30 μg. The selection of antibiotics was based on the recommendations in the CLSI guidelines.^30^

### Determination of resistance genes using PCR

The selection of isolates for resistance-gene determination was based on the resistance profiles to the different antibiotic classes of interest. *E. coli* isolates that were resistant to co-trimoxazole (*n* = 70), ciprofloxacin (*n* = 40), cefotaxime (*n* = 17) and imipenem (*n* = 2) were screened for sulphamethoxazole/trimethoprim-resistance genes, plasmid-mediated quinolone-resistant (PMQR) genes, ESBL/*ampC*, and carbapenemase-resistance genes, respectively. The primer selection and PCR protocol were as earlier described by Farkas *et al.*^31^ Fifty-eight enterococci that were resistant to macrolides were screened for macrolide-resistance genes. The primer selection and PCR protocol were based on those by Zou *et al.*^32^

### Data management and analysis

Questionnaire data were collected using the CSPro 7.6, while the AST data were entered into WHONET 2020. The data were then managed in Excel spreadsheets and exported to STATA14 for analysis. The proportion (%) of resistant, intermediate, susceptible and MDR, XDR, pan-drug resistant (PDR) isolates were estimated with WHONET analysis. MDR isolates were defined as resistance to at least one agent in three or more antibiotic classes, XDR as resistance to at least one agent in all but two or fewer antimicrobial categories (i.e. bacterial isolates remained susceptible to only one or two categories) and PDR was defined as resistance to all agents in all antimicrobial categories.^33^ The data on prescribing patterns were analysed using the IBM Statistical Package for Social Sciences version 25.0 and presented in tables and figures.

### Ethics

The study was conducted according to the guidelines of the Declaration of Helsinki and approved by the Ethics Committee at Eres Converge institutional review board (Ref. No. 2019-Aug-017). The regulatory approval was obtained from the National Health Research Authority (NHRA). Permission to conduct the study at the different hospitals/institutions was obtained from the provincial and district health directors of all the healthcare facilities included in the study. Written informed consent was sought and obtained from the participants before administering the oral questionnaires and collecting samples; for paediatric patients, their guardians gave consent. Only those that gave consent were included. The study participants were assured of confidentiality by not identifying them by name but by codes, age and sex. The data were secured, and the results were used for research purposes only. To ensure safety, the collection of swabs was performed by a qualified health professional.

## Results

Six health facilities were enrolled in this study, three from the Lusaka district (Chilenje, Kalingalinga and Kanyama hospitals) and three from the Ndola district (Lubuto, Mapalo and Masala health centres).

### Prescribing patterns in primary healthcare facilities

**Figure 1.**
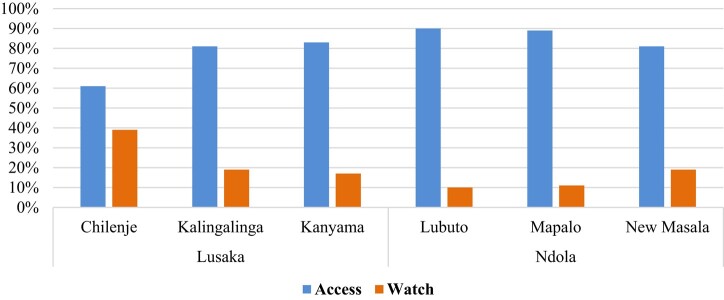
Antibiotic prescriptions based on AWaRe classification of the primary healthcare facilities in Lusaka and Ndola districts.

Most antibiotics prescribed in the primary healthcare facilities in the Lusaka and Ndola districts belonged to the Access group of antibiotics (Table 1). Chilenje first-level hospital had the highest ceftriaxone prescribed (Table 1). The top four most commonly prescribed antibiotics were amoxicillin, cefalexin, metronidazole and co-trimoxazole, with amoxicillin and cefalexin being the most commonly prescribed in Lusaka and Ndola primary healthcare facilities, respectively (Tables 1 and 2). Ciprofloxacin was the most commonly prescribed Watch antibiotic at all primary healthcare facilities (Tables 1 and 2). All the primary healthcare facilities adhered to the WHO AWaRe framework of having ≥60% of prescribed antibiotics belonging to the Access group of antibiotics. Prescription of the Watch group of antibiotics was below 20% in five primary healthcare facilities, with only Chilenje recording 39% (Figure 1).

**Table 1.** Prescribing patterns at primary healthcare facilities in Lusaka district

Name of antibiotics	Indication	Frequency (*n*)	Percent (%)	AWaRe classification
Amoxicillin	RTI, otitis media	7272	23	Access
Metronidazole	UTI, diarrhoea	5871	18	Access
Ciprofloxacin	UTI, PID, STI	4955	15	Watch
Cefalexin	RTI, UTI, pyelonephritis	4873	15	Access
Co-trimoxazole	Diarrhoea, prophylaxis for HIV patients	2242	7	Access
Cefotaxime/Ceftriaxone	RTI, UTI, sepsis	1792	6	Watch
Azithromycin	RTI, COVID-19-related symptoms, tonsillitis	1469	5	Watch
Doxycycline	UTI, STI	1284	4	Access
Benzathine penicillin	Tonsillitis, syphilis	882	3	Access
Gentamicin	STI	751	2	Access
Cloxacillin	RTI	378	1	Access
Penicillin V	URTI, tonsillitis	289	1	Access
Nitrofurantoin	UTI	134	0	Access
Chloramphenicol	Otitis media	25	0	Access
Ampicillin	Sepsis	8	0	Access
Total	—	32 205	100	—

RTI, respiratory tract infection; URTI, upper RTI; STI, sexually transmitted infection; PID, pelvic inflammatory Disease.

**Table 2.** Prescribing patterns at health primary healthcare facilities in Ndola district

Name of antibiotic	Indication	Frequency (*n*)	Per cent (%)	AWaRe classification
Cefalexin	RTI, UTI	7038	20	Access
Amoxicillin	RTI	5834	17	Access
Metronidazole	Diarrhoea	5558	16	Access
Co-trimoxazole	URTI, RTI	5403	16	Access
Ciprofloxacin	UTI	3182	9	Watch
Cloxacillin	RTI, wounds, burns	1830	5	Access
Penicillin	Febrile illness, sepsis	1634	5	Access
Doxycycline	UTI	1416	4	Access
Azithromycin/erythromycin	RTI	1338	4	Watch
Gentamicin	UTI	411	1	Access
Chloramphenicol	Conjunctivitis	89	0	Access
Total	—	34 594	100	—

**Figure 2.**
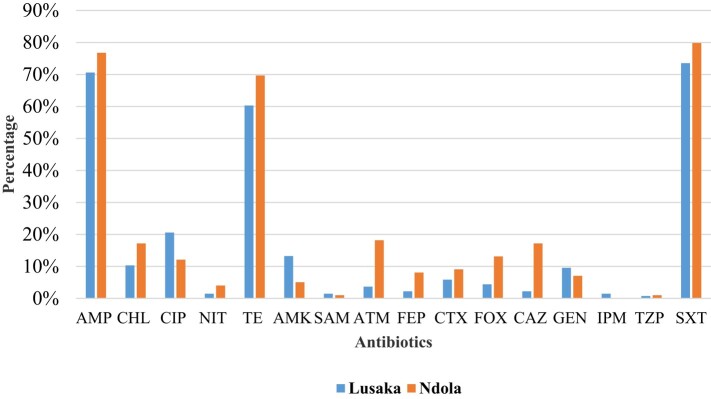
Resistance profiles of *E. coli* from healthy individuals at primary healthcare facilities in Lusaka and Ndola districts. AMP, ampicillin; CHL, chloramphenicol; CIP, ciprofloxacin; NIT, nitrofurantoin; TE, tetracycline; AMK, amikacin; SAM, ampicillin/sulbactam; ATM, aztreonam; FEP, cefepime; CTX, cefotaxime; FOX, cefoxitin; CAZ, ceftazidime; GEN, gentamicin; IPM, imipenem; TZP, piperacillin/tazobactam; SXT, co-trimoxazole.

The prevalence of *E. coli* and *Enterococcus* species was 94% (272/290) and 69% (200/290), respectively. AMR in *E. coli* was highest in sulfamethoxazole/trimethoprim (co-trimoxazole) (76%), ampicillin (73%) and tetracycline (64%) and lowest in imipenem (1%) and piperacillin/tazobactam (1%) (Figure 2). Resistance in *E. coli* was generally higher in Ndola compared with Lusaka (Figure 2).

**Figure 3.**
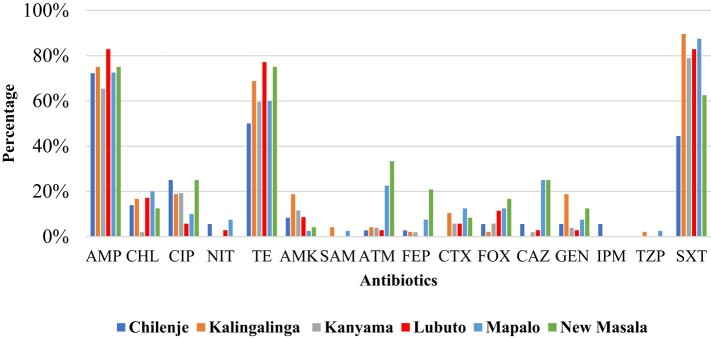
Resistance profiles for *E. coli* from the different primary healthcare facilities in Lusaka and Ndola districts. Lusaka primary healthcare facilities—Chilenje, Kalingalinga, Kanyama; Ndola primary healthcare facilities—Lubuto, Mapalo, New Masala. AMP, ampicillin; CHL, chloramphenicol; CIP, ciprofloxacin; NIT, nitrofurantoin; TE, tetracycline; AMK, amikacin; SAM, ampicillin/sulbactam; ATM, aztreonam; FEP, cefepime; CTX, cefotaxime; FOX, cefoxitin; CAZ, ceftazidime; GEN, gentamicin; IPM, imipenem; TZP, piperacillin/tazobactam; SXT, co-trimoxazole.

Resistance to ampicillin was noted to be high in all six different healthcare facilities. Similarly, co-trimoxazole and tetracycline resistance were high in all the facilities, with the least resistance in Chilenje. Imipenem resistance was only seen in Chilenje, while piperacillin/tazobactam and ampicillin/sulbactam resistance was recorded in Mapalo (Ndola) and Kalingalinga (Lusaka) health facilities. Resistance to other antibiotics such as chloramphenicol, ciprofloxacin, ceftriaxone, gentamicin, amikacin and aztreonam was recorded in all the primary healthcare facilities but at diverse percentages (Figure 3).

**Figure 4.**
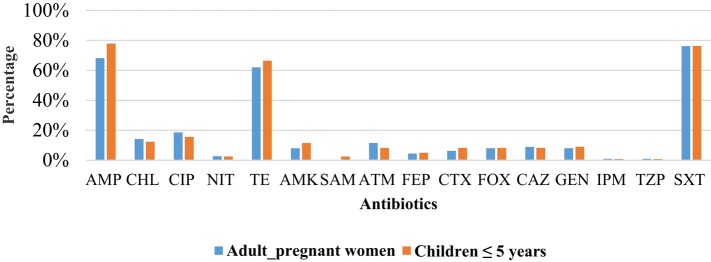
Resistance profiles of *E. coli* from healthy adults (pregnant women) and children ≤5 years old. AMP, ampicillin; CHL, chloramphenicol; CIP, ciprofloxacin; NIT, nitrofurantoin; TE, tetracycline; AMK, amikacin; SAM, ampicillin/sulbactam; ATM, aztreonam; FEP, cefepime; CTX, cefotaxime; FOX, cefoxitin; CAZ, ceftazidime; GEN, gentamicin; IPM, imipenem; TZP, piperacillin/tazobactam; SXT, co-trimoxazole.

There was no difference in the occurrence of AMR in adults (pregnant women) compared with children ≤5 years old (Figure 4) for all antibiotics tested except for amikacin (*P* = 0.033) and co-trimoxazole (*P* = 0.032). Though low, resistance to antibiotics such as ceftriaxone, cefepime, piperacillin/tazobactam and amikacin, which are used to treat invasive infections in hospitalized patients, was noted in both pregnant women and children ≤5 years old (Figure 4).

### Resistance-gene determinants in E. coli

Several resistance-gene determinants were identified (Table 3). Notably, most isolates had multiple resistance genes from the same class and/or from other antibiotic classes. The prevalence of ESBL resistance-gene determinants was 5% (14/272), co-trimoxazole 39% (70/179) and fluoroquinolones 7% (20/272).

**Table 3.** Resistance-gene determinants in *E. coli*

Antibiotic class	Resistance genes	Frequency, % (*n*)
β-Lactams		
** **ESBL resistance-gene determinants	*bla*_SHV_	48 (11)
	*bla*_TEM_	30 (7)
	*bla*_CTX-M_	22 (5)
** **Total		100 (23)
Folate-pathway antagonists		
** **Sulphamethoxazole	*Sul2*	52 (43)
	*sul1*	26 (21)
** **Trimethoprim	*dfA7*	22 (18)
** **Total		100 (82)
Fluoroquinolones		
	*qnrA*	36 (19)
	*qnrS*	34 (18)
	*qnrB*	30 (16)
** **Total		100 (53)

### Enterococci from a healthy community

Among the identified enterococci*, E. faecium* was predominant compared with *E. faecalis* and the other species, which were in relatively low numbers (Table 4).

**Table 4.** The distribution of *Enterococcus* species from the healthy individuals in the community

*Enterococcus* species	Frequency (*n*)	Percent (%)
*E. faecium*	93	46
*E. faecalis*	85	43
*E. gallinarum*	10	5
*E. hirae*	8	4
*E. casseliflavus*	2	1
*E. durans*	2	1
Total	200	100

**Figure 5.**
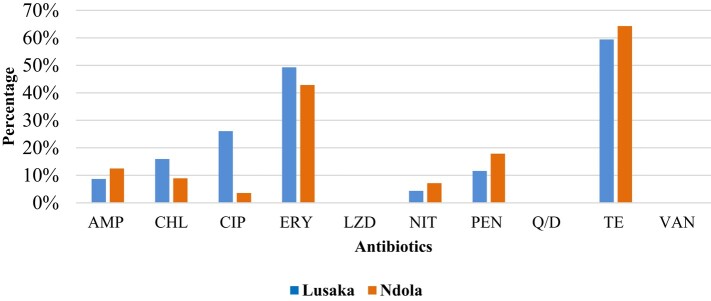
Resistance profiles for enterococci from healthy communities in Lusaka and Ndola districts. AMP, ampicillin; CHL, chloramphenicol; CIP, ciprofloxacin; ERY, erythromycin; LZD, linezolid; NIT, nitrofurantoin, PEN, penicillin, Q/D, quinupristin/dalfopristin; TE, tetracycline; VAN, vancomycin.

The highest resistance was observed to tetracycline, followed by erythromycin for both Lusaka and Ndola primary healthcare facilities (Figure 5). There was no resistance recorded to linezolid, quinupristin/dalfopristin and vancomycin, though reduced susceptibility (intermediate results) were as follows: linezolid (*n* = 31), quinupristin/dalfopristin (*n* = 16) and vancomycin (*n* = 14). These three antibiotics are used to treat invasive infections and are considered last-resort treatment options for MDR *Enterococcus* infections.

**Figure 6.**
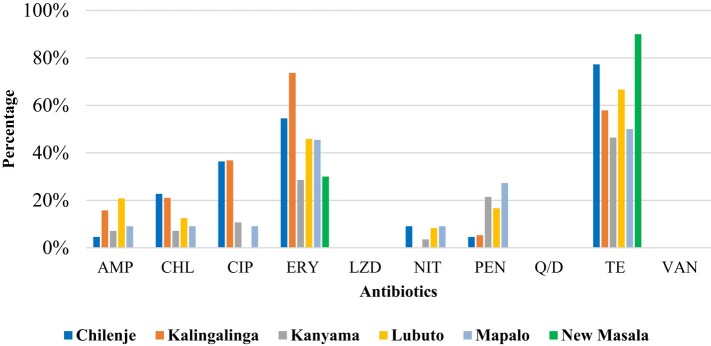
Resistance profiles for enterococci from the different primary healthcare facilities in Lusaka and Ndola districts. AMP, ampicillin; CHL, chloramphenicol; CIP, ciprofloxacin; ERY, erythromycin; LZD, linezolid; NIT, nitrofurantoin, PEN, penicillin, Q/D, quinupristin/dalfopristin; TE, tetracycline; VAN, vancomycin.

Erythromycin resistance was highest at Kalingalinga, followed by Chilenje, while tetracycline resistance was highest at New Masala, followed by Chilenje. The three primary healthcare facilities in the Lusaka district recorded the highest frequency of reduced susceptibility to linezolid, quinupristin/dalfopristin and vancomycin (Figure 6).

**Figure 7.**
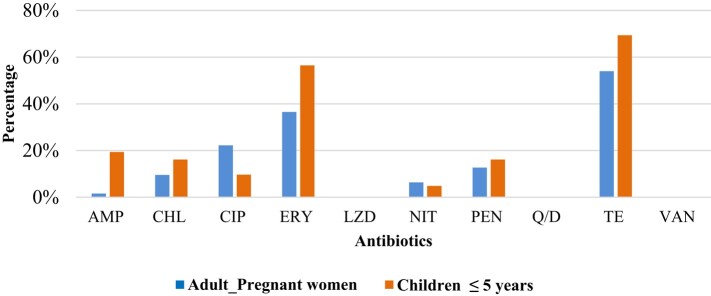
Resistance profiles in adults (pregnant women) and children ≤5 years old. AMP, ampicillin; CHL, chloramphenicol; CIP, ciprofloxacin; ERY, erythromycin; LZD, linezolid; NIT, nitrofurantoin, PEN, penicillin, Q/D, quinupristin/dalfopristin; TE, tetracycline; VAN, vancomycin.

The reduced susceptibility to the three antibiotics was mostly in adult pregnant women than children ≤5 years old. Resistance to tetracycline, erythromycin, nitrofurantoin and ciprofloxacin was higher in adult pregnant women than in children ≤5 years old, while resistance to the remaining antibiotics tested was higher in children ≤5 years old than in adult pregnant women (Figure 7). In children ≤5 years old, reduced susceptibility to linezolid was found in 10, to quinupristin/dalfopristin in 9 and to vancomycin in 5.

Resistance to macrolides was 29% (58/200), and the prevalence of macrolide-resistance genes was 16% (32/200), with *erm*(B) (69%; 22/32) being the most prevalent, followed by *erm*(C) (19%; 6/32) and *erm*(A) (12%; 4/32).

Seventy-five per cent (153/204) of *E. coli* and 25% (51/204) of enterococci were classified as MDR, with Kalingalinga and Kanyama primary healthcare facilities recording the highest MDR *E. coli* and enterococci rates, respectively. XDR was more prevalent in enterococci isolates (97%; 29/30) compared with *E. coli* (3%; 1/30). Only one *Enterococcus* isolate was classified as PDR (Table 5).

**Table 5.** Distribution of MDR, XDR and PDR in *E. coli* and *Enterococcus* isolates

Location	Facility	Frequency, *n* (%)		
		MDR	XDR	PDR
*E. coli*				
** **Lusaka	Chilenje	25 (16)	—	—
	Kalingalinga	34 (22)	—	—
	Kanyama	24 (16)	1 (100)	—
** **Ndola	Lubuto	28 (18)		
	Mapalo	22 (15)		
	Masala	20 (13)		
** **Total (*E. coli*)		153 (75)	1 (3)	0
Enterococci				
** **Lusaka	Chilenje	11(21.5)	8 (28)	—
	Kalingalinga	5 (10)	4 (14)	—
	Kanyama	13 (25)	10 (34)	1 (100)
** **Ndola	Lubuto	12 (23.5)	4 (14)	—
	Mapalo	5 (10)	2 (7)	—
	Masala	5 (10)	1(3)	
** **Total (Enterococci)		51 (25)	29 (97)	1 (100)
Total (*E. coli* and enterococci)		204 (100)	30 (100)	1 (100)

## Discussion

This study assessed the prescribing patterns of antibiotics and AMR profiles of carriage *E. coli* and enterococci isolated from pregnant women and children ≤5 years old in the Lusaka and Ndola districts of Zambia. Our study found that the Access group of antibiotics, namely amoxicillin, metronidazole, cefalexin and co-trimoxazole, were the most commonly prescribed in the primary healthcare facilities, similar to findings in two districts in Cameroon that both recorded amoxicillin, co-trimoxazole and metronidazole as the most commonly prescribed.^34^ Penicillins were widely used in primary healthcare, with oral amoxicillin being the most commonly prescribed; comparable to our findings, a scoping review found amoxicillin to be the most commonly prescribed in East Africa, West Africa, New Zealand, Cuba and Brazil,^35^ and India.^36^ The overuse of penicillins in primary healthcare facilities could be attributed to the lack of facility-based treatment guidelines supported by local microbiology data.^14^

Although the prescription of antibiotics was above the WHO-recommended threshold of 30%, the choice of antibiotic classes in the primary healthcare facilities was in line with the WHO recommendations, which state that more than 60% of all prescribed antibiotics must be from the Access group.^18,37^ Our findings agreed with those from a study that included Ghana, Uganda, Tanzania and Zambia, where almost half of the antibiotics prescribed belonged to WHO’s Access group.^38^ Ceftriaxone, ciprofloxacin and erythromycin/azithromycin were the only prescribed antibiotics belonging to the WHO Watch group, with ciprofloxacin being the most commonly prescribed. Similar to our finding, a study at nine primary healthcare facilities in India found ciprofloxacin to be the most prescribed Watch antibiotic.^36^ Contrary to our findings, a study that assessed antibiotic overuse in primary healthcare settings revealed higher use of Watch antibiotics, mostly quinolones and cephalosporins, in China and India, with only Kenya mostly using Access antibiotics.^39^ A study in rural Burkina Faso that assessed the use of Watch antibiotics before hospital presentation and another study in China that assessed antibiotic prescription patterns in 48 primary healthcare facilities both found ceftriaxone to be the most commonly prescribed, followed by ciprofloxacin.^40,41^ The limited availability of culture and sensitivity testing (CST) to guide antibiotic choices in primary healthcare facilities results in consistently high rates of empirical prescribing.^42^

Overprescribing of antibiotics in primary healthcare facilities has resulted in AMR, limiting the treatment options and increasing the population carriage of resistant organisms in the community.^43^ Encouragingly, none of the facilities included in this study prescribed antibiotics from the WHO Reserve group, and the three antibiotics from the Watch group accounted for less than 20% in all facilities except one that also serves as a first-level hospital. This was contrary to a study by D’Arcy *et al.*^38^ that found the use of Watch antibiotics to be above 38% in all four studied countries, with Zambia recording 41% use. Again, contrary to our findings, a study in India recorded the use of Reserve antibiotics such as carbapenems and colistin, the last-resort antibiotics for treating MDR, XDR and ESBL-producing Gram-negative bacilli, respectively.^39,44^ Inappropriate prescribing of Watch and Reserve antibiotics outside specialist hospitals reduces their potential to tackle serious and critical infections when needed.^45^

High levels of *E. coli* resistance to ampicillin and co-trimoxazole could be attributed to selective pressure resulting from misuse and overuse of antibiotics in the community and at primary healthcare facilities.^46,47^ Comparable to our findings, a systematic review that analysed the prevalence of resistance to the top 10 antibiotics commonly prescribed in LMICs in commensal *E. coli* isolates from human sources in community settings found high resistance to ampicillin, co-trimoxazole and tetracycline.^48^ Studies included in this systematic review were mostly from Asia (*n* = 13) and Africa (*n* = 10).^48^ The use of co-trimoxazole for prophylaxis of opportunistic infections in people living with HIV (PLWHIV) might also promote selective pressure, hence the high levels of resistance noted.^49^ A study in southern Ethiopia revealed that co-trimoxazole prophylaxis increased the risk of resistance to co-trimoxazole, and this was statistically associated with co-resistance to penicillin.^49^

High antibiotic resistance rates were observed in carriage *E. coli* and enterococci from healthy individuals from the communities, especially to antibiotics commonly accessed without prescriptions and used as empirical treatment in primary health facilities.^22,46^ This agrees with the rising resistance in healthy individuals, especially children, who are potential reservoirs of antibiotic-resistant bacteria.^50^ Studies have linked carriage of antibiotic-resistant organisms in healthy communities to poor sanitation and infrastructure in high-density areas, environmental contamination, high prevalence of HIV in LMICs and the lack of regulations that allow access to antibiotics without an indication and prescriptions.^51–55^ In this study, antibiotic resistance was recorded in both high- and medium-density areas, which could be attributed to the behaviour towards antibiotic use in the community and the lack of knowledge on the effects of irrational antibiotic use.^56,57^

The presence of resistance genes found on mobile genetic elements such as plasmids is alarming as these can easily be transferred to other bacteria that can potentially cause invasive infections.^58^ Notably, ESBL resistance genes (*bla*_CTX-M_, *bla*_SHV_, *bla*_TEM_), PMQR genes (*qnrA*, *qnrS* and *qnrB*) and trimethoprim/sulphamethoxazole-resistance genes (*sul1*, *sul2*, *dfA7*) recorded in healthy individuals in this study were also found in clinical isolates causing BSIs at the University Teaching Hospital, a tertiary hospital in Lusaka, Zambia.^59^ A study in uMgungundlovu, South Africa, revealed a higher prevalence of ESBL-mediating MDR Gram-negative ESKAPE bacteria in faecal carriage (46%) than in clinical samples (28%), with colonization being mainly associated with a referral from district to tertiary hospitals.^60^

The presence of MDR and XDR in carriage *E. coli* and enterococci in this study further increases the chances of transmitting resistance genes to pathogenic bacteria, which might lead to increased transfer of resistant pathogens in the community and hospital settings.^61^ The rise in MDR/XDR pathogens causing serious illnesses limits treatment options, prolongs hospital stay, increases treatment costs and leads to poor treatment outcomes.^62^ Most patients who seek primary healthcare facility services present with respiratory tract infections and acute diarrhoea, which could be caused by viruses and/or be self-limiting.^63^ However, due to the lack of diagnostic capacity in these facilities and poor knowledge of AMR, these patients are treated with antibiotics.^16,64,65^ Habits such as purchasing half the course, self-medication, sharing medicines and interruption of treatment may contribute to resistance and the development of MDR/XDR in the community.^56^ The need to inform and educate the communities on the drivers of AMR and their effects and to implement measures that tackle inappropriate use and behavioural change cannot be overemphasized.^65^

This study was conducted during the COVID-19 pandemic, which could explain the high resistance to erythromycin as an attribute of self-medication and the irrational use of macrolides during the COVID-19 pandemic.^66^ The other limitations were the inability to speciate enterococci and screen for more resistance genes due to financial constraints. Lastly, antimicrobial prescribing data were extracted from paper-based databases, which had some limitations in that not all the files had complete data and the records were not up to date.

### Conclusions

Antibiotic prescribing patterns in primary healthcare facilities adhered to the WHO AWaRe framework. However, carriage *E. coli* and enterococci from healthy individuals were mostly resistant to the prescribed antibiotics. These findings highlight the need to use local susceptibility data to formulate country-specific treatment guidelines in line with the WHO Aware classification. The high resistance and MDR to affordable antibiotics are a public health concern requiring urgent actions such as improving diagnostic capacity and surveillance, strengthening antimicrobial stewardship programmes, enforcing regulations that forbid easy access to antibiotics, and community AMR education and awareness.
